# Supplementary material for: Causal Relationship between Aging and Anorexia Nervosa: A White-Matter-Microstructure-Mediated Mendelian Randomization Analysis
Source: Biomedicines. 2024 Aug 16;12(8):1874. doi: 10.3390/biomedicines12081874 (PMC11351342; doi:10.3390/biomedicines12081874)
Supplement: Supplementary file 1 [file biomedicines-12-01874-s001.zip › Supplementary Figure/Figure S8/Leaveoneout plot of MR analysis for TL to ubm-b-1525.pdf]

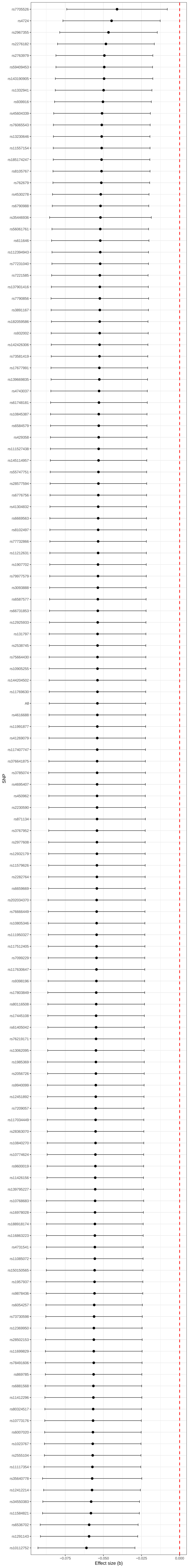
rs7705526
rs4724
rs2967355
rs2276182
rs2763979
rs59409453
rs143190905
rs1332941
rs939916
rs45604339
rs76065543
rs13230646
rs11557154
rs185174247
rs8105767
rs762679
rs4530278
rs6790988
rs35446936
rs56061761
rs611646
rs112394943
rs77231040
rs7221585
rs137901416
rs7790856
rs3891167
rs182059586
rs932002
rs142426306
rs73581419
rs17677991
rs139669835
rs4743037
rs61748181
rs10645387
rs6584579
rs429358
rs111527438
rs145114957
rs55747751
rs28577594
rs6776756
rs41304832
rs6669563
rs8102497
rs77732866
rs11212631
rs1907702
rs79977579
rs3093888
rs6587577
rs66731853
rs12925933
rs131797
rs2538745
rs75664430
rs10905255
rs144204502
rs11769630
All
rs4616688
rs11991877
rs41269079
rs117407747
rs376641875
rs3785074
rs4695407
rs450962
rs2230590
rs871134
rs3767952
rs2977608
rs12932179
rs11579626
rs2282764
rs6659669
rs202034370
rs76666449
rs10805346
rs111950327
rs117512405
rs7099229
rs117630647
rs9398196
rs17803849
rs80116508
rs17445108
rs61405042
rs76219171
rs13062095
rs1985369
rs2056726
rs9940099
rs12451892
rs7209057
rs117034449
rs28363070
rs10840270
rs10774624
rs9600019
rs11426156
rs139795227
rs10768683
rs16978028
rs188918174
rs116863223
rs4731541
rs11085072
rs150150565
rs1957937
rs9878436
rs6054257
rs73730598
rs12369950
rs28502153
rs11699829
rs78491606
rs869785
rs6881568
rs11412296
rs80324517
rs10773176
rs6007020
rs1023767
rs2555104
rs11117354
rs35640778
rs12412214
rs34550383
rs11584821
rs6536702
rs1291143
rs10112752
SNP
−0.075
−0.050
−0.025
0.000
Effect size (b)
